# Annotations

**Published:** 1892-11-26

**Authors:** 


### Annotations.

Is the London Medical School a Failure?

The London Medical School, Professor Erichsen considers, ought to be the finest Imperial medical school in the world. It is, as a matter of fact, a mere provincial institution. If this did not make Mr. Erichsen unhappy, it would say little for Mr. Erichsen. With every resource of teachers, teaching material, and students, London, according to the Principal of University College, must be written down a failure. We cannot quote Mr. Erichsen's pamphlet in full—would that we could! Nor can we give anything like an adequate selection of its facts and arguments. But, summed up, they amount to this—that the London Medical School has been a failure in the past, is increasingly a failure in the present, and ought to be the most magnificent success in the world. We will not take upon ourselves to endorse the professor's sweeping charges of failure; but the latter portion of his proposition we agree with entirely. London has the teachers, the teaching material, and the students to furnish forth the completest and most splendid medical school the world has ever seen. Where is the fault? It lies with the medical teachers—with them and with no one else. Every hospital, every teacher in every hospital has fought for his own hand: and hence has resulted disorganisation, confusion, inefficiency, and stark failure. The London student who is cleverer than his fellow goes to Vienna or to Berlin to learn what he cannot be taught in the greatest metropolis in the world, and in medical schools manned by Englishmen. This is a great humiliation, which our professional brethren inflict upon our country, our city, and ourselves. Mr. Erichsen proposes to mend matters by federating the hospitals into one great school, and to this end he issues a pamphlet. That is well as a beginning, and we would like to see the pamphlet spread broadcast. But as soon as it shall have done its work—say, in three months—Mr. Erichsen should proceed to the formation of a small, practical, and disinterested committee. There is a prodigious work to be done before the London hospitals can be brought to the mood of federation. If Mr. Erichsen wishes to see anything practical accomplished in his lifetime, let him leave pamphleteering and put his hand to the plough.

"Long runs."

Mr. W. C. Sidgwick, writing to the *Times* from Rugby, makes assertions about headmasters and certain kinds of athletic exercises that are said to be compulsory upon public schoolboys of a very disquieting character. "Long runs" at school have their value for some boys. But it would be just as reasonable to insist that every boy should be six feet high as to insist that he is a fit and proper person to take long runs. The hearts of boys have to be taken into consideration when selecting their athletic exercises; so, too, have their lungs and nervous systems. Now, the hearts of persons differ in every respect quite as much as their noses or other features do; they differ in size, in muscular strength, in nervous force, in power of endurance. It is precisely the same with their lungs and nervous systems. And, if we would but think of it, this is exactly what one would naturally expect. Why should there be more uniformity of shape and capacity among internal organs than among external ones? As a matter of fact there is not. Yet Mr. Sidgwick tells us that "Everybody, young and old, strong and weakly, who is not actually ill, is compelled by the authority of the headmaster to join in these runs." Well, if this be indeed so, it is very astonishing, but it is proper to say that it has since been contradicted. At Rugby, to take the school already under discussion, the traditions of headmastership ought to be of the very quintessence of reason, science, and good sense. There never lived a more reasonable man than Dr. Arnold, and he has not been so long dead but that many memories and practical fruits of his influence must still survive. We ask headmasters this question: Is it not reasonable to suppose that doctors and physiologists are fitter judges of the capacity of boys for physical exercise than headmasters? Would not any headmaster be justly incensed if a doctor of medicine should presume to claim superiority over him in the classical languages and antiquities? The thing is obvious. Here and there may be a doctor who is generally uncultured, and even stupid, in his own profession. But the average doctor, however poorly he may compare with the average headmaster in literary culture, not only ought to know, but does know, infinitely more about all questions of physiology and health. We must come to reason in this matter. Physiology consists largely of assured knowledge nowadays, and the physiologist cannot sit still in silence whilst he sees foolish and cruel things done on schoolboys which serve no purpose but one which is entirely harmful. Such silence is both cowardly and wrong. Even boys at public schools ought to have reasonable protection against ignorance in authority.

Riotous Living at Kidderminster Workhouse.

The story which comes to us from Kidderminster to the effect that the "drink bill" for its 332 paupers last year amounted to something over £240 is almost incredible. But the one fact in connection with the story which makes belief most difficult is that it is not the medical officer who has turned on the beer tap so liberally, but the guardians. There are only two explanations of this unparalleled piece of parochial history which strike us as coming within the bounds of possibility. The guardians must be either lunatics or brewers. If a medical officer had done this thing, there is not a board of guardians in the kingdom but would have declared that he richly deserved to be hanged. To get at the true enormity of the performance we have to compare the amount spent in drink at the Kidderminster Union with that spent at similar unions elsewhere. But first let this fact be noted, that whilst the average annual amount spent on stimulants for paupers throughout Great Britain and Ireland is 1s. 8½d. per head, at Kidderminster it is 12s. 2¼d. per head. Compare now the Kidderminster pauper with his fellow-pauper at Aston, a neighbouring union. At Aston the pauper gets beer, and no doubt quite as much as is good for him. But his fellow at Kidderminster gets exactly 73 times as much, or more in a week than the other gets in a year. There are some whole counties in England—nay, even some groups of small counties—which spend less in drink on their paupers than Kidderminster does on its single union. The medical officer last year prescribed no more stimulants than amounted in cost to £30. The guardians, on their own sole responsibility, increased this amount eightfold. What is to be done? Has the Local Government Board the power and the will to disestablish the guardians? Fanatical teetotalers might propose to drown the whole of them in a beer vat; but that would be too strong a first measure. Perhaps a beginning might be made with the chairman, and so terror would be struck into the rest. But, seriously, this is a grave scandal. If the guardians do not immediately put an end to it, the Local Government Board must suspend or dismiss them. Alas! for the shade of Richard Baxter, that even the paupers of his parish should be tempted by their natural protectors to live riotously in the very workhouse!

